# HLA polymorphisms and risk of glioblastoma in Koreans

**DOI:** 10.1371/journal.pone.0260618

**Published:** 2021-12-09

**Authors:** Sang-Soo Choi, Haeyoun Choi, In-Cheol Baek, Soon A. Park, Jae-Sung Park, Tai-Gyu Kim, Sin-Soo Jeun, Stephen Ahn

**Affiliations:** 1 Department of Neurosurgery, Seoul St. Mary’s Hospital, College of Medicine, The Catholic University of Korea, Seoul, South Korea; 2 Department of Microbiology, College of Medicine, The Catholic University of Korea, Seoul, South Korea; 3 Catholic Hematopoietic Stem Cell Bank, College of Medicine, The Catholic University of Korea, Seoul, South Korea; 4 Department of Biomedicine and Health Sciences, College of Medicine, The Catholic University of Korea, Seoul, South Korea; Jordan University of Science and Technology, JORDAN

## Abstract

**Purpose:**

Immune responses for cancer cells can be altered according to genetic variation of human leukocyte antigen (HLA). Association of HLA polymorphism with risk of various cancer types is well known. However, the association between HLA and glioblastoma (GBM) remains uncertain. We sought to evaluate the association of HLA polymorphism with risk of GBM development in Koreans.

**Materials and methods:**

A case-control study was performed to identify the odds ratios (OR) of HLA class I and II genes for GBM. The control group consisted of 142 healthy Korean volunteers, and the GBM group was 80 patients with newly diagnosed GBM at our institution. HLA class I (-A, -B, and–C) and class II (-DR, -DQ, and–DP) genotyping was performed by high-resolution polymerase chain reaction (PCR)-sequence-based typing (PCR-SBT) methods.

**Results:**

There were significantly decreased frequencies of HLA-A*26:02 (OR 0.22 CI 0.05–0.98), HLA-C*08:01 (OR 0.29 CI 0.10–0.87), and HLA-DRB1*08:03 (OR 0.32 CI 0.11–0.98), while there was significantly increased frequency of HLA-C*04:01 (OR 2.29 CI 1.05–4.97). In analysis of haplotypes, the frequency of DRB1*14:05-DQB1*05:03 was significantly decreased (OR 0.22 CI 0.05–0.98).

**Conclusion:**

This study suggests that genetic variations of HLA may affect GBM development in Koreans. Further investigations with larger sample sizes are needed to delineate any potential role of the HLA polymorphisms in the pathogenesis of GBM development.

## Introduction

Glioblastoma (GBM) is the most common and most fatal primary malignant brain tumor in adults. Its prognosis is devastating, with median survival of 14.6 months and 2-year survival less than 30%, despite aggressive multimodal treatment including surgical resection, concomitant chemoradiation (CCRT), and adjuvant temozolomide chemotherapy [1,2]. Numerous researchers have sought to identify risk factors associated with glioma development to inhibit genesis and progression of this tumor; however, the only established findings were that history of radiation increased the risk of gliomas [3,4], while history of allergies and autoimmune diseases decreased the risk of gliomas [5–7].

Human leukocyte antigen (HLA), a surface molecule expressed in most human cells and encoded by the major histocompatibility complex (MHC) gene complex in humans, has a major role of presenting antigenic peptides to T lymphocytes and modulating immune responses [8,9]. HLA genes are highly polymorphic and can induce different immune responses according to genetic variation [10,11]. In addition, these polymorphisms are related with risk of allergies and autoimmune disease and with various cancers including Hodgkin’s lymphoma, leukemia, cervical cancer, nasopharyngeal cancer, and lung cancer [9,12–16].

A few studies have evaluated potential association between HLA polymorphism and risk of glioma; however, the results remain unclear [17–24]. Most studies used serologic methods, which resulted in only two-digit resolution of HLA genotypes [19–23]. Also, all but one study included only western populations [18].

In this context, we evaluated the potential association between HLA polymorphisms and risk of GBM development in Koreans by comparing four-digit resolution of HLA genotypes of normal individuals (control group) with those of GBM patients (GBM group). As molecular characteristics vary by GBM type, we included GBM without isocitrate dehydrogenase (IDH) mutation or 1p/19q co-deletion.

## Materials and methods

### Patient and control groups

This study was approved by the Institutional Review Board (IRB) of Seoul St. Mary’s Hospital (KC18TESI0224). Korean patients with newly diagnosed GBM who were present for follow-up at the Neuro-oncology Center of Seoul St. Mary’s Hospital from March 2018 to December 2019 were included as the patient group after providing informed consent. The informed consent was written on the document. The diagnosis was confirmed by a neuropathologist according to the 2016 World Health Organization Classification of Tumors of Central Nervous System (CNS). We only included newly diagnosed primary GBM, not secondary or recurrent GBM. Patients with previous history of other cancers or autoimmune diseases were excluded. IDH 1 mutation was evaluated by immunohistochemistry or directing sequencing, and 1p/19q co-deletion was detected using fluorescence in situ hybridization (FISH). O [6]-Methylguanine-DNA methyltransferase (MGMT) gene methylation status was evaluated by polymerase chain reaction (PCR). Status of survival and/or date of death were obtained from the Korea Central Cancer Registry database. Overall survival (OS) was defined as days from initial surgery to death, and progression-free survival (PFS) was defined as days from initial surgery to progression confirmed by magnetic resonance image, according to the immunotherapy response assessment for neuro-oncology criteria. Patients alive on August 31, 2020, were censored. The average duration of follow-up was 15.0 months (range 2–115 months).

The control group consisted of 142 healthy Korean volunteers who were genetically unrelated to one other under control of the IRB. The mean age of the control group was 30 years, and the proportion of males was 50.7% (males 72 and females 70).

### DNA extraction and HLA genotyping

After we received informed consent from patients, we acquired 4ml of peripheral blood for DNA extraction. Genomic DNA was extracted from this peripheral blood mixed with ethylenediaminetetraacetic acid according to standard methods using TIANamp Genomic DNA Extraction Kits (Tiangen Biotech Corporation, Beijing, China), according to the manufacturer’s instructions.

The genotyping of HLA class I (-A, -B, and–C) and class II (-DR, -DQ, and–DP) was performed using polymerase chain reaction-sequence-based typing (PCR-SBT) methods, as described in a previous study [25]. In total, 20 alleles for HLA-A, 36 alleles for HLA-B, 22 alleles for HLA-C, 29 alleles for HLA-DRB1, 16 alleles for HLA-DQB1, and 13 alleles for HLA-DPB1 were detected. Amino acid sequences for HLA-A, -B, -C, -DRB1, -DQB1, and -DPB1 with a resolution of 4 digits were obtained from the international ImMunoGeneTics references [26]. Analysis of variant amino acids was performed across all HLA-A, -B, -C, -DRB1, -DQB1, and -DPB1 alleles present in the genotyping results.

### Statistical analysis

All clinical variables were characterized in a descriptive manner. Kaplan-Meier survival analysis and the log-rank test were used to estimate median OS and PFS. The difference in the frequencies of HLA alleles in control and GBM groups was compared using the Chi-square test or Fisher’s exact test. Odds ratio (OR) and 95% confidence interval (CI) were estimated by logistic regression; when zero samples were observed, logistic regression using Firth’s bias reduction was applied. Statistical analysis was performed using SAS software ver. 9.4 (SAS Institute, Cary, NC, United States). P-values < 0.05 and CIs that did not cross 1 were considered statistically significant.

## Results

### Baseline characteristics of GBM patients

A total of 80 patients who met the eligibility criteria was included. In total, 45 (56.3%) were male and 35 (43.8%) were female. The median age of the patient group was 63.0 (range 20–81) years. Almost all patients (90%) did not exhibit IDH1 mutation or 1p/19q co-deletion; in the other 10% of patients, these features were undetermined. MGMT promoter was methylated in 52.5% patients. Median progression-free survial and overall survival were 8.5 months (range 2–87 months) and 18.0 (4–87 months), respectively. Baseline characteristics of the GBM group are described in Table 1.

**Table 1:** Clinical characteristics of the GBM group.

Characteristics	GBM group (n = 80)
Male sex, n (%)	45 (56.3%)
Median age, years (range)	63.0 (20–81)
IDH1 mutation, n (%)	
Yes	0 (0%)
No	72 (90.0%)
Unknown	8 (10.0%)
1p/19q co-deletion, n (%)	
Yes	0 (0%)
No	72 (90.0%)
Unknown	8 (10.0%)
MGMT methylation, n (%)	
Yes	42 (52.5%)
No	38 (47.5%)
Unknown	0 (0%)
Median progression-free survival, months (range)	8.5 (2–87)
Median overall survival, months (range)	18.0 (4–87)

### Associations of HLA Class I and II alleles with GBM risk

In HLA Class I, the HLA-A*26:02 (2.5% vs. 10.6% p = 0.030, OR 0.22 CI 0.05–0.98) and HLA-C*08:01 (5.0% vs.15.5% p = 0.020, OR 0.29 CI 0.10–0.87) allele frequencies were significantly lower in the GBM group (Table 2). Also, the HLA-C*04:01 (20.0% vs. 9.9%, p = 0.034, OR 2.29 CI 1.05–4.97]) allele frequency was significantly higher in the GBM group (Table 2). In contrast, the HLA-A*30:04 (0.0% vs. 6.3% p = 0.028, OR 0.09, CI 0.00–1.77) and the HLA-B*40:06 (0.0% vs. 7.0%, OR 0.08, CI 0.00–1.56) frequencies had p-values below 0.05, but ORs that did not indicate statistical significance. In HLA Class II, HLA-DRB1*08:03 (5.0% vs. 14.1% p = 0.036, OR 0.32 CI 0.11–0.98) was significantly lower in the GBM group. HLA-DQB1*0503 (5.0% vs. 13.4%, p = 0.049, OR 0.34, CI 0.11–1.04) had a p-value below 0.05, but an OR that did not indicate statistical significance (Table 3).

**Table 2:** HLA Class I allele frequencies in GBM and control groups.

Alleles	GBM group (n = 80), n (%)		Control group (n = 142), n (%)		*P* value	Odds ratio (95% CI)
**A**						
01:01	5	(6.3)	4	(2.8)	0.289	-
02:01	23	(28.8)	53	(37.3)	0.196	-
02:03	2	(2.5)	5	(3.5)	> 0.999	-
02:06	15	(18.8)	24	(16.9)	0.728	-
02:07	7	(8.8)	8	(5.6)	0.374	-
03:01	4	(5.0)	2	(1.4)	0.192	-
11:01	19	(23.8)	33	(23.2)	0.931	-
11:02	0	(0.0)	1	(0.7)	> 0.999	-
24:02	28	(35.0)	46	(32.4)	0.693	-
26:01	5	(6.3)	9	(6.3)	0.979	-
26:02	2	(2.5)	15	(10.6)	0.030	0.22 (0.05–0.98)
26:03	2	(2.5)	1	(0.7)	0.295	-
29:01	2	(2.5)	0	(0.0)	0.129	-
30:01	7	(8.8)	12	(8.5)	0.939	-
30:04	0	(0.0)	9	(6.3)	0.028	0.09 (0.00–1.77)
31:01	7	(8.8)	11	(7.7)	0.793	-
32:01	1	(1.3)	1	(0.7)	> 0.999	-
33:03	22	(27.5)	38	(26.8)	0.905	-
33:25	1	(1.3)	0	(0.0)	0.360	-
68:01	1	(1.3)	0	(0.0)	0.360	-
**B**						
07:02	7	(8.8)	13	(9.2)	0.919	-
07:05	2	(2.5)	13	(1.4)	0.058	-
08:01	1	(1.3)	2	(1.4)	> 0.999	-
13:01	5	(6.3)	4	(2.8)	0.289	-
13:02	8	(10.0)	13	(9.2)	0.836	-
14:01	0	(0.0)	7	(4.9)	0.051	-
15:01	20	(25.0)	22	(15.5)	0.082	-
15:07	0	(0.0)	3	(2.1)	0.555	-
15:11	1	(1.3)	3	(2.1)	> 0.999	-
15:18	2	(2.5)	5	(3.5)	> 0.999	-
27:04	0	(0.0)	1	(0.7)	> 0.999	-
27:05	7	(8.8)	10	(7.0)	0.646	-
35:01	11	(13.8)	15	(10.6)	0.478	-
35:03	1	(1.3)	0	(0.7)	0.360	-
37:01	5	(6.3)	5	(3.5)	0.502	-
38:02	2	(2.5)	6	(4.2)	0.714	-
39:01	0	(0.0)	2	(1.4)	0.537	-
40:01	4	(5.0)	10	(7.0)	0.548	-
40:02	6	(7.5)	9	(6.3)	0.741	-
40:03	2	(2.5)	2	(1.4)	0.621	-
40:04	1	(1.3)	0	(0.0)	0.360	-
40:06	0	(0.0)	10	(7.0)	0.015	0.08 (0.00–1.56)
44:02	3	(3.8)	3	(2.1)	0.670	-
44:03	14	(17.5)	19	(13.4)	0.407	-
46:01	5	(6.3)	16	(11.3)	0.220	-
48:01	3	(3.8)	15	(10.6)	0.074	-
51:01	8	(10.0)	24	(16.9)	0.160	-
51:02	1	(1.3)	3	(2.1)	> 0.999	-
52:01	7	(8.8)	5	(3.5)	0.124	-
54:01	5	(6.3)	20	(14.1)	0.076	-
55:02	1	(1.3)	6	(4.2)	0.426	-
56:01	1	(1.3)	1	(0.7)	> 0.999	-
57:01	1	(1.3)	1	(0.7)	> 0.999	-
58:01	11	(13.8)	16	(11.3)	0.587	-
59:01	5	(6.3)	3	(2.1)	0.140	-
67:01	3	(3.8)	4	(2.8)	0.705	-
**C**						
01:02	25	(31.3)	48	(33.8)	0.697	-
01:03	0	(0.0)	3	(2.1)	0.555	-
02:02	2	(2.8)	1	(0.7)	0.295	-
03:02	11	(13.8)	16	(11.3)	0.587	-
03:03	14	(17.5)	28	(19.7)	0.843	-
03:04	12	(15.0)	21	(14.8)	0.685	-
04:01	16	(20.0)	14	(9.9)	0.034	2.29 (1.05–4.97)
05:01	3	(4.1)	3	(2.1)	0.670	-
06:02	14	(17.5)	18	(12.7)	0.326	-
07:01	1	(1.3)	0	(0.0)	0.360	-
07:02	16	(20.0)	25	(17.6)	0.659	-
07:04	2	(2.9)	5	(3.5)	> 0.999	-
07:06	1	(1.5)	6	(4.2)	0.426	-
08:01	4	(5.0)	22	(15.5)	0.020	0.29 (0.10–0.87)
08:02	0	(0.0)	7	(4.9)	0.051	-
08:03	0	(0.0)	3	(2.1)	0.555	-
12:02	7	(8.9)	6	(4.2)	0.233	-
12:03	1	(1.8)	2	(1.4)	> 0.999	-
14:02	9	(11.5)	21	(14.8)	0.459	-
14:03	12	(15.0)	15	(10.6)	0.332	-
15:02	3	(3.8)	8	(5.6)	0.750	-
15:05	2	(2.5)	8	(5.6)	0.336	-

**Table 3:** HLA Class II allele frequencies in GBM and control groups.

Locus	GBM group (n = 80), n (%)		Control group (n = 142), n (%)		*P* value	Odds ratio (95% CI)
**DRB1**						
01:01	11	(13.8)	20	(14.1)	0.945	-
03:01	4	(5.0)	8	(5.6)	> 0.999	-
04:01	2	(2.5)	2	(1.4)	0. 621	-
04:03	3	(3.8)	8	(5.6)	0.750	-
04:04	2	(2.5)	6	(4.2)	0.714	-
04:05	15	(18.8)	16	(11.3)	0.123	-
04:06	8	(10.0)	11	(7.7)	0.564	-
04:07	0	(0.0)	3	(2.1)	0.555	-
04:08	1	(1.3)	0	(0.0)	0.360	-
04:10	0	(0.0)	3	(2.1)	0.555	-
07:01	11	(13.8)	19	(13.4)	0.938	-
08:02	6	(7.5)	10	(7.0)	0.899	-
08:03	4	(5.0)	20	(14.1)	0.036	0.32 (0.11–0.98)
09:01	9	(11.3)	23	(16.2)	0.314	-
10:01	3	(3.8)	4	(2.8)	0.705	-
11:01	8	(10.0)	12	(8.5)	0.699	-
11:06	0	(0.0)	1	(0.7)	> 0.999	-
12:01	6	(7.5)	9	(6.3)	0.741	-
12:02	8	(10.0)	10	(7.0)	0.438	-
13:01	4	(5.0)	2	(1.4)	0.192	-
13:02	18	(22.5)	23	(16.2)	0.245	-
14:03	3	(3.8)	2	(1.4)	0.354	-
14:04	0	(0.0)	1	(0.7)	> 0.999	-
14:05	4	(5.0)	15	(10.6)	0.155	-
14:06	1	(1.3)	4	(2.8)	0.656	-
14:54	5	(6.3)	6	(4.2)	0.531	-
15:01	12	(15.0)	21	(14.8)	0.966	-
15:02	8	(10.0)	13	(9.2)	0.836	-
16:02	2	(2.5)	5	(3.5)	> 0.999	-
**DQB1**						
02:01	4	(5.0)	7	(4.9)	> 0.999	-
02:02	10	(12.5)	17	(12.0)	0.908	-
03:01	26	(32.5)	35	(24.6)	0.208	-
03:02	15	(18.8)	30	(21.1)	0.672	-
03:03	11	(13.8)	25	(17.6)	0.454	-
03:04	1	(1.3)	0	(0.0)	0.360	-
04:01	14	(17.5)	17	(12.0)	0.254	-
04:02	5	(6.3)	14	(9.9)	0.356	-
05:01	18	(22.5)	26	(18.3)	0.452	-
05:02	6	(7.5)	7	(4.9)	0.553	-
05:03	4	(5.0)	19	(13.4)	0.049	0.34 (0.11–1.04)
06:01	10	(12.5)	27	(19.0)	0.211	-
06:02	12	(15.0)	21	(14.8)	0.966	-
06:03	4	(5.0)	2	(1.4)	0.102	-
06:04	12	(15.0)	16	(11.3)	0.421	-
06:09	7	(8.8)	8	(5.6)	0.374	-
**DPB1**						
01:01	1	(1.3)	0	(0.0)	0.360	-
02:01	33	(41.3)	64	(45.1)	0.582	-
02:02	6	(7.5)	8	(5.6)	0.583	-
03:01	7	(8.8)	7	(4.9)	0.261	-
04:01	15	(18.8)	22	(15.5)	0.532	-
04:02	13	(16.3)	21	(14.8)	0.772	-
05:01	40	(50.0)	88	(62.0)	0.083	-
09:01	6	(7.5)	9	(6.3)	0.741	-
13:01	6	(7.5)	19	(13.4)	0.183	-
14:01	2	(2.5)	5	(3.5)	> 0.999	-
17:01	3	(3.8)	9	(6.3)	0.544	-
38:01	1	(1.3)	0	(0.0)	0.360	-
47:01	0	(0.0)	1	(0.7)	> 0.999	-

### Associations of haplotypes frequencies with GBM risk

In analysis of haplotypes, the frequency of DRB1*14:05-DQB1*05:03 (2.5% vs. 10.6%, p = 0.030, OR 0.22, CI 0.05–0.98) was significantly decreased compared with that of the control group (Table 4). In contrast, the frequency of DRB1*08:03-DQB1*06:01 (5.0% vs. 13.4%, p = 0.049, OR 0.34, CI 0.11–1.04) and the frequency of DRB1*08:03-DQB1*06:01-DPB1*05:01 (2.5% vs. 9.5%, p = 0.042, OR 0.24, CI 0.05–1.06) had p-values below 0.05, but ORs that did not indicate statistical significance (Table 4).

**Table 4:** Genetic influence of HLA 2 and 3 locus haplotypes in glioblastoma patients.

HLA haplotype alleles	GBM group (n = 80), n (%)		Control group (n = 142), n (%)		*P* value	Odds ratio (95% CI)
**2-locus haplotypes**						
DRB1*08:03-DQB1*06:01	4	(5.0)	19	(13.4)	0.049	0.34 (0.11–1.04)
DRB1*14:05-DQB1*05:03	2	(2.5)	8	(10.6)	0.030	0.22 (0.05–0.98)
**3-locus haplotypes**						
DRB1*08:03-DQB1*06:01-DPB1*05:01	2	(2.5)	7	(9.5)	0.042	0.24 (0.05–1.06)

## Discussion

Avoiding of immunologic surveillance of cancer cells is a well-established mechanism for oncogenesis, and T cells have been suggested to have a major role in immune systems to control cancer cells [27,28]. Many researchers have found that T cell-mediated immune responses against cancer cells were significantly different within individuals, and the main reasons for this heterogeneous response were due to polymorphisms of immune-related genes such as HLA genes [29,30]. According to genetic variations of HLA genes, the ability of MHC to present tumor-associated antigens can be different, as can responses between antigen-loaded MHC and T cell receptor (TCR) of T cells [31]. These differences can affect T cell-mediated immune responses against cancer cells, which is related with disease susceptibility and prognosis in cancer patients [9]. Numerous studies have found that genetic variations of HLA were responsible for different immune responses for tumor-associated antigens of cancer cells and were strongly related with susceptibility to several cancers [9,12–15,17,18,30,32].

While CNS was considered an immune-privileged system, a few studies have evaluated the potential association between genetic variation of HLA genes and susceptibility to glioma development (Table 5). These studies have several limitations. First, except for two recent studies [17,18], all studies used PCR-sequence-specific primers (PCR-SSP) or serologic methods and could identify only two-digits of resolution of HLA genotypes. Second, the HLA alleles suggested to be related with increased or decreased risk of glioma were not identical between studies. In two studies, HLA-A*25, B*27, DRB1*15, DRB1*07, and DQB1*06 were commonly found as alleles related with risk of glioma. The recent largest study including more than 1000 people of European ancestry showed positive association between haplotype of DRB1*15:01-DQA1*01:02-DQB1*06:02 and risk of glioma development [17]. These results were consistent with those of two previous studies including Caucasians that showed increased glioma risk in people with HLA-DRB1*15. However, another recent study including Northern Chinese citizens showed decreased risk of glioma development in people with HLA-A*0201. Third, all but one study included only western populations [18]. This discrepancy may be explained by several possible issues. Gliomas are composed of heterogeneous subgroups such as astrocytoma, oligodendroglioma, and glioblastoma, which have distinct mechanisms of gliomagenesis. Heterogeneity of grade and subtype in a disease group may affect associations between HLA and the risk of glioma. In addition, there may have been bias regarding control group selection. Further studies to compensate for various confounders such as heterogeneity of glioma and selection of study populations are needed.

**Table 5:** Summary of studies evaluating associations between genetic variations of HLA and glioma development.

Year	Authors	Population	Disease	Case	Control	Genotyping	Results
This Study	Stephen Ahn et al	Koreans	Glioblastoma	80	152	PCR-SBT	***Increased*** : C*04:01 ***Decreased*** : A*26:02 : C*08:01 : DRB1*08:03 : DRB1*14:05-DQB1*05:03
2017	Zhang C et al	European ancestry	Glioma	1,746	2,312	Next-generation sequencing	***Increased*** DRB1*15:01-DQA1*01:02-DQB1*06:02
2017	Han S et al	Northern China	Glioma	150	150	PCR-SBT	***Decreased*** A*02:01
2011	Bassig BA et al	European ancestry	Glioma	340	255	PCR-SSP	***Increased*** DQB1*06 DRB1*13 ***Decreased*** DQB1*05
2009	Song W et al	European American	Glioblastoma	149	149	PCR-SSP	***Increased*** : Cw*05 ***Decreased*** : A*32 : B*14 : B*40
2009	La Torre D et al	Sicily	Glioma	56	140	PCR-SSP	***Increased*** A*11 DQB1*06 DRB1*14 ***Decreased*** : B*07 : C*04
2006	Guerini FR et al	Northern Italy	Glioma	36	71,945^a^/97^b^/2,054^c^	PCR-SSP	***Increased*** : DRB1*14
2005	Tang J et al	Caucasian	Glioblastoma	155	157	PCR-SSP	***Increased*** : A*24 : A*25 : B*27 : DRB1*15 **Decreased** : DRB1*07 : Cw*06-DRB1*07
2001	Machulla HK et al	Caucasian	Glioma	65	157	PCR or Serologic methods	***Increased*** : A*25 : B*27 : DRB1*15 : DRB1*15-DRB5*(51) ***Decreased*** : DRB1*07 : Cw*6-DRB1*07

^a^71,945 healthy controls for HLA-A and -B type.

^b^97 healthy controls for HLA-DQB1 type.

c2,045 cadaver organ donors for HLA-DRB1 type.

In this context, we sought to evaluate whether the polymorphism of HLA can affect the risk of GBM development in Koreans. We used the methods of PCR-SBT and achieved four-digit resolution of HLA genotypes of normal Korean’s individuals (control group) and those of GBM patients (GBM group). In this study, we showed that the frequencies of HLA-A*26:02 (OR 0.22 CI 0.05–0.98), HLA-C*08:01 (OR 0.29 CI 0.10–0.87), and HLA-DRB1*08:03 (OR 0.32 CI 0.11–0.98) were significantly decreased, while the frequency of HLA-C*04:01 (OR 2.29 CI 1.05–4.97) was significantly increased. Analysis of haplotypes and frequencies showed that DRB1*14:05-DQB1*05:03 was significantly decreased in the GBM group (OR 0.22 CI 0.05–0.98). Our findings support a study evaluating association of HLA genes with autoimmune disease in Koreans. While the history of autoimmune disease was considered as a protective factor for glioma development, previous study found increased frequency of HLA-DRB1*08:03 and HLA-DRB1*08:03-DQB1*06:01 in populations with autoimmune thyroid disease. In this study, HLA-DRB1*08:03 (OR 0.32 CI 0.11–0.98) was significantly related to decreased risk for GBM development; haplotype HLA-DRB1*08:03-DQB1*06:01 was also related to decreased risk of GBM development, although the OR was not statistically significant (OR 0.34 0.11–1.04).

To the best of our knowledge, our study firstly has found positive associations between the polymorphisms of HLA and IDH-wildtype GBM in an East-Asian population. We tried to include homogeneous patients confirmed by both histological and molecular features according to 2016 WHO classifications to reduce selection bias. In addition, as an era of immunotherapy, this landscape of HLA polymorphisms in GBM patients may provide more understanding of immunotherapies focusing on TCR-peptide/MHC interactions such as peptide vaccines, neoantigen vaccines, and TCR-engineered T cell therapy. Further preclinical and clinical investigations are needed to delineate any potential role of the HLA polymorphisms in the pathogenesis of GBM development. We hope that in this era of immunotherapy, this information on HLA polymorphisms in GBM patients may provide insight into immunotherapies focusing on TCR-peptide/MHC interactions such as peptide vaccines, neoantigen vaccines, and TCR-engineered T cell therapy.

Our findings should be considered with several limitations. First, our findings were not consistent previous studies, although almost previous studies include only Europeans, and the HLA composition of Europeans was different from that of Koreans. Second, we could not exclude the possibility of linkage disequilibrium with other unmeasured alleles in the region in glioma development. Third, we did not adjust all factors potentially that influence hazard ratios such as sex, age, and several undefined factors. Also, there could be some biases for the control group, which included 142 healthy Korean volunteers. Fourth, the suggested alleles associated with GBM susceptibility showed low frequencies, such as HLA-A*26:02 (2.5%), which may have led to biased findings. Fifth, subtype analysis including the subtype of GBM, such as pronerual, neural, classical, or mesenchymal, could not be performed in this study. Further research that includes GBM subtype is needed. Lastly, we could not perform multiple tests for confirm the significance of p-value, which need much larger patient’s samples.

### Conclusions

This study suggests that genetic variations of HLA may affect the risk of GBM development in Koreans. Further investigations with larger sample sizes are needed to delineate any potential role of the HLA polymorphisms in the pathogenesis of GBM development.

## Supporting information

S1 Data (XLSX)

## References

[1] Stupp R, Hegi ME, Mason WP, Van Den Bent MJ, Taphoorn MJ, Janzer RC, et al. Effects of radiotherapy with concomitant and adjuvant temozolomide versus radiotherapy alone on survival in glioblastoma in a randomised phase III study: 5-year analysis of the EORTC-NCIC trial. The lancet oncology. 2009;10(5):459–66. doi: 10.1016/S1470-2045(09)70025-7 19269895

[2] Stupp R, Mason WP, Van Den Bent MJ, Weller M, Fisher B, Taphoorn MJ, et al. Radiotherapy plus concomitant and adjuvant temozolomide for glioblastoma. New England journal of medicine. 2005;352(10):987–96. doi: 10.1056/NEJMoa043330 15758009

[3] Ostrom QT, Adel Fahmideh M, Cote DJ, Muskens IS, Schraw JM, Scheurer ME, et al. Risk factors for childhood and adult primary brain tumors. Neuro-oncology. 2019;21(11):1357–75. doi: 10.1093/neuonc/noz123 31301133 PMC6827837

[4] Ostrom QT, Bauchet L, Davis FG, Deltour I, Fisher JL, Langer CE, et al. The epidemiology of glioma in adults: a “state of the science” review. Neuro-oncology. 2014;16(7):896–913. doi: 10.1093/neuonc/nou087 24842956 PMC4057143

[5] Wiemels JL, Wiencke JK, Sison JD, Miike R, McMillan A, Wrensch M. History of allergies among adults with glioma and controls. International journal of cancer. 2002;98(4):609–15. doi: 10.1002/ijc.10239 11920623

[6] Pouchieu C, Raherison C, Piel C, Migault L, Carles C, Fabbro-Perray P, et al. Allergic conditions and risk of glioma and meningioma in the CERENAT case-control study. Journal of Neuro-Oncology. 2018;138(2):271–81. doi: 10.1007/s11060-018-2816-6 29500663

[7] Disney-Hogg L, Cornish AJ, Sud A, Law PJ, Kinnersley B, Jacobs DI, et al. Impact of atopy on risk of glioma: a Mendelian randomisation study. BMC medicine. 2018;16(1):1–13. doi: 10.1186/s12916-018-1027-5 29540232 PMC5853158

[8] Parham P, Ohta T. Population biology of antigen presentation by MHC class I molecules. Science. 1996;272(5258):67–74. doi: 10.1126/science.272.5258.67 8600539

[9] Dendrou CA, Petersen J, Rossjohn J, Fugger L. HLA variation and disease. Nature Reviews Immunology. 2018;18(5):325. doi: 10.1038/nri.2017.143 29292391

[10] Probst C, Bompeixe E, Pereira N, et al., De O. Dalalio M, Visentainer J, et al. HLA polymorphism and evaluation of European, African, and Amerindian contribution to the white and mulatto populations from Paraná, Brazil. Human Biology. 2000:597–617. 11048789

[11] Carey BS, Poulton KV, Poles A. Factors affecting HLA expression: A review. International journal of immunogenetics. 2019;46(5):307–20. doi: 10.1111/iji.12443 31183978

[12] Fletcher LB, Veenstra RN, Loo EY, Hwang AE, Siddiqi IN, Visser L, et al. HLA expression and HLA type associations in relation to EBV status in Hispanic Hodgkin lymphoma patients. PLoS One. 2017;12(3):e0174457. doi: 10.1371/journal.pone.0174457 28334025 PMC5363938

[13] Yoon J. Acute myeloid leukemia is a disease associated with HLA-C3. Acta haematologica. 2015;133(2):164–7. doi: 10.1159/000365436 25278127

[14] Kamiza AB, Kamiza S, Mathew CG. HLA-DRB1 alleles and cervical cancer: A meta-analysis of 36 case-control studies. Cancer epidemiology. 2020;67:101748. doi: 10.1016/j.canep.2020.101748 32562888

[15] Yang H, Yu K, Zhang R, Li J, Wei X, Zhang Y, et al. The HLA-DRB1 allele polymorphisms and nasopharyngeal carcinoma. Tumor Biology. 2016;37(6):7119–28. doi: 10.1007/s13277-016-5051-9 27059731

[16] Li Y, Liu S, Hong C, Ma Q, Tan F, Liu C, et al. The association of HLA/KIR genes with non-small cell lung cancer (adenocarcinoma) in a Han Chinese population. Journal of Cancer. 2019;10(20):4731. doi: 10.7150/jca.33566 31598144 PMC6775512

[17] Zhang C, de Smith AJ, Smirnov IV, Wiencke JK, Wiemels JL, Witte JS, et al. Non-additive and epistatic effects of HLA polymorphisms contributing to risk of adult glioma. Journal of neuro-oncology. 2017;135(2):237–44. doi: 10.1007/s11060-017-2569-7 28721485 PMC5665694

[18] Han S, Deng J, Wang Z, Liu H, Cheng W, Wu A. Decreased human leukocyte antigen A* 02: 01 frequency is associated with risk of glioma and existence of human cytomegalovirus: a case–control study in Northern China. Cancer Immunology, Immunotherapy. 2017;66(10):1265–73. doi: 10.1007/s00262-017-2018-7 28523518 PMC11028914

[19] Bassig BA, Inskip PD, Burdette L, Shapiro WR, Selker RG, Fine HA, et al. Selected human leukocyte antigen class II polymorphisms and risk of adult glioma. Journal of neuroimmunology. 2011;233(1–2):185–91. doi: 10.1016/j.jneuroim.2010.11.005 21195488 PMC3074044

[20] Song W, Ruder AM, Hu L, Li Y, Ni R, Shao W, et al. Genetic epidemiology of glioblastoma multiforme: confirmatory and new findings from analyses of human leukocyte antigen alleles and motifs. PLoS One. 2009;4(9):e7157. doi: 10.1371/journal.pone.0007157 19774073 PMC2742900

[21] La Torre D, Maugeri R, Angileri FF, Pezzino G, Conti A, Cardali SM, et al. HUMAN LEUKOCYTE ANTIGEN FREQUENCY IN HUMAN HIGH‐GRADE GLIOMAS: A CASE‐CONTROL STUDY IN SICILY. Neurosurgery. 2009;64(6):1082–9. doi: 10.1227/01.NEU.0000345946.35786.92 19487887

[22] Guerini FR, Agliardi C, Zanzottera M, Delbue S, Pagani E, Tinelli C, et al. Human leukocyte antigen distribution analysis in North Italian brain Glioma patients: an association with HLA-DRB1* 14. Journal of Neuro-oncology. 2006;77(2):213. doi: 10.1007/s11060-005-9032-x 16314951

[23] Tang J, Shao W, Dorak MT, Li Y, Miike R, Lobashevsky E, et al. Positive and negative associations of human leukocyte antigen variants with the onset and prognosis of adult glioblastoma multiforme. Cancer Epidemiology and Prevention Biomarkers. 2005;14(8):2040–4. doi: 10.1158/1055-9965.EPI-05-0136 16103458

[24] Machulla HK, Steinborn F, Schaaf A, Heidecke V, Rainov NG. Brain glioma and human leukocyte antigens (HLA)–is there an association. Journal of neuro-oncology. 2001;52(3):253–61. doi: 10.1023/a:1010612327647 11519856

[25] Shin D-H, Baek I-C, Kim HJ, Choi E-J, Ahn M, Jung MH, et al. HLA alleles, especially amino-acid signatures of HLA-DPB1, might contribute to the molecular pathogenesis of early-onset autoimmune thyroid disease. PloS one. 2019;14(5):e0216941. doi: 10.1371/journal.pone.0216941 31091281 PMC6519818

[26] Lefranc M-P, Giudicelli V, Duroux P, Jabado-Michaloud J, Folch G, Aouinti S, et al. IMGT®, the international ImMunoGeneTics information system® 25 years on. Nucleic acids research. 2015;43(D1):D413–D22. 25378316 10.1093/nar/gku1056 PMC4383898

[27] Hanahan D, Weinberg RA. Hallmarks of cancer: the next generation. cell. 2011;144(5):646–74. doi: 10.1016/j.cell.2011.02.013 21376230

[28] Restifo NP, Dudley ME, Rosenberg SA. Adoptive immunotherapy for cancer: harnessing the T cell response. Nature Reviews Immunology. 2012;12(4):269–81. doi: 10.1038/nri3191 22437939 PMC6292222

[29] Garrido F. HLA Class-I Expression and Cancer Immunotherapy. MHC Class-I Loss and Cancer Immune Escape: Springer; 2019. p. 79–90. 10.1007/978-3-030-17864-2_3 31140107

[30] Garrido F, Aptsiauri N. Cancer immune escape: MHC expression in primary tumours versus metastases. Immunology. 2019;158(4):255–66. doi: 10.1111/imm.13114 31509607 PMC6856929

[31] Sewell AK. Why must T cells be cross-reactive? Nature Reviews Immunology. 2012;12(9):669–77. doi: 10.1038/nri3279 22918468 PMC7097784

[32] Kim H-J, Choi H-B, Jang J-P, Baek I-C, Choi E-J, Park M, et al. HLA-Cw polypmorphism and killer cell immunoglobulin-like receptor (KIR) gene analysis in Korean colorectal cancer patients. International Journal of Surgery. 2014;12(8):815–20. doi: 10.1016/j.ijsu.2014.06.012 24998207

